# Patient Outcomes in Resected Combined Hepatocellular Cholangiocarcinoma (cHCC-ICC) and Intrahepatic Cholangiocarcinoma: A Single Center Study

**DOI:** 10.3390/cancers16223878

**Published:** 2024-11-20

**Authors:** Rick Y. Lin, Doga Kahramangil, Muhammet Ozer, Thomas J. George, Ibrahim Nassour, Steven J. Hughes, Ali Zarrinpar, Ilyas Sahin

**Affiliations:** 1 Department of Medicine, College of Medicine, University of Florida, Gainesville, FL 32610, USA; ricklin@medicine.ufl.edu; 2 Department of Medicine, Division of Hematology and Oncology, College of Medicine, University of Florida, Gainesville, FL 32610, USA; dogakahramangilb@ufl.edu (D.K.); thom.george@medicine.ufl.edu (T.J.G.); 3 Department of Medical Oncology, Dana Farber Cancer Institute, Harvard Medical School, Boston, MA 02115, USA; muhammet_ozer@dfci.harvard.edu; 4 Department of Surgery, University of Florida, Gainesville, FL 32610, USA; ibrahim.nassour@surgery.ufl.edu (I.N.); steven.hughes@surgery.ufl.edu (S.J.H.); ali.zarrinpar@surgery.ufl.edu (A.Z.); 5 Massachusetts General Hospital Cancer Center, Harvard Medical School, Boston, MA 02114, USA

**Keywords:** combined hepatocellular cholangiocarcinoma (cHCC-ICC), intrahepatic cholangiocarcinoma (ICC), surgical resection, recurrence-free survival, overall survival

## Abstract

Background/Objectives: Combined hepatocellular cholangiocarcinoma (cHCC-ICC) is a rare malignancy that involves a combination of features of hepatocellular carcinoma and intrahepatic cholangiocarcinoma (ICC) and exhibits a more aggressive clinical course; however, its risk factors and outcomes remain largely undefined. Methods: This study is a single-center retrospective study of 82 patients diagnosed with ICC or cHCC-ICC who underwent surgical resection from June 2011 to January 2023. Our analysis included 70 patients with resected ICC and 12 with resected cHCC-ICC. Results: The overall survival (OS) for the entire cohort was 21.6 months, with a recurrence-free survival (RFS) of 11.8 months. The cHCC-ICC group had significantly higher levels of AST and ALT (AST median 206 U/L vs. 46 U/L; ALT median 165.5 U/L vs. 48 U/L; *p* = 0.012 and *p* = 0.013, respectively), whereas the ICC group had higher alkaline phosphatase (median 66 U/L vs. 104 U/L; *p* = 0.03). CA 19-9 values (76 U/mL vs. 22 U/mL; *p* = 0.02) were higher in the ICC group, while AFP values were higher in the cHCC-ICC group (7.3 ng/mL vs. 3.2 ng/mL; *p* = 0.0004). The cHCC-ICC group had a significantly higher rate of recurrence (83% vs. 47%, *p* = 0.028) with a significantly decreased RFS (4.7 months vs. 12.4 months; log-rank *p* = 0.007). In multivariate analysis, patients with resected ICC had a significantly reduced risk of recurrence by 73% compared to their counterparts (HR 0.27 [0.10–0.73], *p* = 0.01). Conclusions: cHCC-ICC is a rare entity that needs to be further studied to improve patient outcomes. Further studies are warranted and may suggest the need for more aggressive initial treatment strategies in patients diagnosed with cHCC-ICC.

## Simple Summary

Combined hepatocellular cholangiocarcinoma (cHCC-ICC) is a rare subtype of primary liver malignancy and involves a combination of features of both hepatocellular carcinoma and intrahepatic cholangiocarcinoma. cHCC-ICC is generally thought to exhibit more aggressive behavior and is usually associated with poor prognosis. However, the risk factors and outcomes of patients diagnosed with cHCC-ICC remain largely undefined. The aim of our study was to compare the clinical characteristics and survival outcomes of patients diagnosed with intrahepatic cholangiocarcinoma and cHCC-ICC who have undergone surgical resection. We conducted a single-center retrospective study with 82 patients. Our analysis showed that the cHCC-ICC group had significantly higher rates of recurrence as well as significantly decreased recurrence-free survival. Our study showed that combined hepatocellular cholangiocarcinoma is a rare entity that needs to be further studied to improve patient outcomes.

## 1. Introduction

Hepatocellular carcinoma (HCC) is the most common primary liver cancer, accounting for around 70% of all cases, followed by intrahepatic cholangiocarcinoma (ICC), which accounts for 15% [1,2]. Combined hepatocellular cholangiocarcinoma (cHCC-ICC) is an extremely rare subtype of a primary liver malignancy which involves a combination of features of HCC and ICC and accounts for 2–3% of liver malignancies [3].

As compared to other malignancies, cHCC-ICC is considered to exhibit more aggressive tumor behavior and usually has a poor prognosis [4,5]. The risk factors and characteristics of a typical patient diagnosed with cHCC-ICC are still largely unknown. One study suggested that the risk factors are highly influenced by geography [6]. This is plausible, considering the well documented variation in HCC incidence across the different regions of the world, particularly between the East and the West, likely due to differences in the prevalence of viral hepatitis [7]. Several studies from East Asia have identified potential risk factors for cHCC-ICC, including heavy alcohol use, hepatitis B infection, male sex, cirrhosis, and diabetes mellitus [8,9].

The clinical diagnosis of cHCC-ICC without a biopsy can be extremely challenging. Several studies have looked retrospectively at tumor markers, including alpha-fetoprotein (AFP), carcinoembryonic antigen (CEA), cancer antigen 19-9 (CA 19-9), as well as imaging and failed to find any unique characteristic features in cHCC-ICC patients [10,11,12]. One retrospective study that was done in the Thai population found in a multivariate analysis that elevated CA 19-9 and intrahepatic bile duct dilation were prognostic for poor survival in cHCC-ICC patients [13]. Additionally, the presentation of cHCC-ICC is similar to that of HCC and ICC. The majority of patients present with generalized symptoms, including fatigue, abdominal pain, jaundice, and weight loss [14]. Patients who present at a more advanced stage may present with more severe symptoms including ascites, hepatomegaly, or acute cholangitis [5].

Treatment modalities for cHCC-ICC depend on the stage of the disease. Several staging criteria originally designed for HCC and ICC, such as the Barcelona Clinic Liver Cancer (BCLC) system and the TNM classification, can be utilized to assess the stage of cHCC-ICC [15,16]. The only curative treatment for patients diagnosed with cHCC-ICC is surgical resection [6]. The role of a liver transplant or other systemic therapies continues to be highly debated in this patient population [17,18]. In fact, one study showed that those who underwent a liver transplant for cHCC-ICC had significantly worse survival compared to transplant patients with HCC [19]. One study looking at surgical outcomes in cHCC-ICC demonstrated that a resection margin of 1 cm or above had better survival [20]. Additionally, another study showed that more aggressive surgical approaches were associated with better survival outcomes [21]. Given the rarity of the disease, the molecular understanding of cHCC-ICC is lacking, which contributes to the scarcity of treatment options [22,23]. Currently, various systemic therapies, such as chemotherapy, targeted therapy, and immunotherapy, are being explored [24]. Despite the perception that cHCC-ICC is typically associated with worse survival given the aggressive nature of the disease, several studies have shown a similar or even better survival of cHCC-ICC compared to that of ICC [12,25,26,27,28]. 

The aim of this study is to compare the clinical characteristics and survival outcomes of patients diagnosed with ICC and cHCC-ICC who have undergone surgical resection in our institution. Our study did not include HCC as it falls outside the scope of our study. 

## 2. Materials and Methods

### 2.1. Study Population

**Figure 1:**
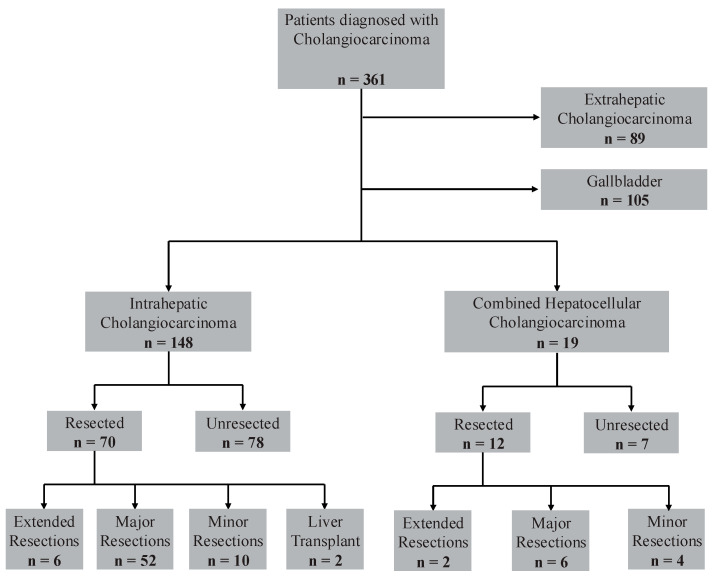
Flowchart for study subject selection.

This was a single-center retrospective study of patients who were either diagnosed with ICC or cHCC-ICC between June 2011 and January 2023. The ICD diagnosis codes used to retrieve data include C22.0, C22.1, C24.0, C23, and C24.9. During this period, there were initially 361 patients diagnosed with cholangiocarcinoma. Patients who were diagnosed with extrahepatic cholangiocarcinoma or gallbladder cancer were excluded. Additionally, patients who did not undergo resection were excluded. Eighty-two patients were included in the final analysis as seen in Figure 1. The study was approved by the University of Florida Institutional Review Board (IRB202300417) and conducted in a manner consistent with the principles of the Declaration of Helsinki. 

### 2.2. Study Variables

This study focused on the clinical outcomes and survival characteristics of patients with cHCC-ICC compared to their counterparts with ICC. This study evaluated various demographic variables, including age, race (White, Black, or Asian), ethnicity (Hispanic, non-Hispanic), and gender (male, female). Additionally, information regarding the patient’s medical history (prior other cancer, history of cirrhosis) and laboratory values at diagnosis (AST, ALT, alkaline phosphatase, total bilirubin, CA 19-9, CEA, AFP, neutrophil/lymphocyte ratio) were obtained. Surgical and pathology data were obtained for all patients, including TNM staging, largest tumor size diameter after resection, presence of lymphovascular and perineural invasion, and type of resection conducted. Recurrence rate and recurrence location were also obtained.

### 2.3. Statistical Analysis

Descriptive statistics were used to create a table to summarize the baseline demographics. Survival data were calculated based on the date of diagnosis until the most recent follow-up date or death from any cause. Mann–Whitney U, Log-rank, Kruskal–Wallis, and Fisher’s tests were used where appropriate. A Cox proportional hazards regression was used to perform a multivariable analysis, adjusting for confounding factors, including age, gender, AST/ALT ratio, ALP, perineural invasion, portal vein invasion, and neutrophil/lymphocyte ratio. A *p*-value of <0.05 was considered statistically significant. Statistical analyses were performed using GraphPad Prism Software version 10.1.1 and SPSS version 22.0.

## 3. Results

### 3.1. Baseline Characteristics 

A total of 82 patients with either resected ICC or cHCC-ICC who were included in our analysis. Baseline patient characteristics are listed in Table 1. The median age at diagnosis was 64, with 52% of patients being male and 48% being female. The majority of patients (94%) were White. Sixteen percent of the patients had a prior diagnosis of another type of malignancy, while 9% had a history of cirrhosis prior to diagnosis. There was a significantly higher number of resected cHCC-ICC who had a history of cirrhosis as compared to the resected ICC group (33% vs. 4%; *p* = 0.008).

### 3.2. Disease Characteristics and Tumor Markers

Baseline tumor characteristics are included in Table 1 as well. The median AST, ALT, and alkaline phosphatase were 62 U/L, 57 U/L, and 87 U/L, respectively. The median total bilirubin was 0.8 mg/dL. The cHCC-ICC groups had significantly higher transaminase levels at the time of diagnosis (AST: 206 vs. 46, *p* = 0.012; ALT: 165.5 vs. 48, *p* = 0.013). On the contrary, alkaline phosphatase was significantly higher in the resected ICC group (104 U/L vs. 66 U/L, *p* = 0.03). For the entire cohort, the median CA 19-9, CEA, and AFP were 64.5 U/mL, 2.4 ng/mL, and 3.8 ng/mL, respectively. In terms of the tumor markers, the resected ICC group had significantly higher CA 19-9 (76 U/mL vs. 22 U/mL, *p* = 0.02), while the cHCC-ICC group had significantly higher AFP levels (7.3 ng/mL vs. 3.2 ng/mL, *p* = 0.0004). 

### 3.3. Surgical Procedural and Histological Data

An overview of surgical interventions is detailed in Table 1 and Figure 1, while histological outcomes after surgical resection can be found in Table 2. Overall, 10% of patients underwent extended resections, 71% underwent major resections, and 17% underwent minor resections. In the ICC group, two patients had liver transplants. The proportions of extended, major, and minor resections were not significantly different between the ICC and cHCC-ICC groups (*p* = 0.173). The median largest resected tumor dimension was 4.8 cm in the ICC group and 5.5 cm in the cHCC-ICC patients. In terms of resection status, most patients in both groups underwent R0 resections with negative margins (86% in ICC and 83% in cHCC-ICC). Six patients in the ICC group had R1 resection status, and four patients had R2 status. In the cHCC-ICC group, two of the twelve patients had R1 resection status. The two groups showed a similar proportion (nearly half) of tumors with lymphovascular invasion. There was a significantly higher percentage of patients in the ICC group with perineural invasion (56% vs. 8%, *p* = 0.007). Details for TNM staging are seen in Table 2.

### 3.4. Neoadjuvant and Adjuvant Treatment 

The majority of patients in the ICC group received surgery alone (60%), while 33% of patients received adjuvant chemotherapy. There were five patients (7%) who received neoadjuvant chemotherapy. Similarly, in the cHCC-ICC cohort, the majority of patients had surgery alone (58%), while 33% had adjuvant chemotherapy. None of the cHCC-ICC patients received neoadjuvant treatment.

### 3.5. Tumor Recurrence

Among all patients, slightly more than half had tumor recurrence (recurrence rate of 52%). The details of the rate of recurrence, as well as the location of recurrence, can be seen in Table 3. There was a significantly higher rate of recurrence in the cHCC-ICC group (83% vs. 47%, *p* = 0.028); however, patterns of recurrence did not seem to differ. In the resected ICC group, 64% had intrahepatic recurrence, 21% had extrahepatic recurrence, and 15% had both intrahepatic and extrahepatic recurrence. Similarly, in the resected cHCC-ICC group, 60% had intrahepatic recurrence, 20% had extrahepatic recurrence, and 20% had both intrahepatic and extrahepatic recurrence. 

In the 33 resected ICC patients who experienced recurrence, 12 (36%) of patients did not undergo further treatment. 21% of patients had chemotherapy alone, and 21% had radiation alone. The remaining 22% of patients either had a combination of chemotherapy, radiation, and immunotherapy or enrolled in a clinical trial. In the resected cHCC-ICC group, three (30%) patients did not undergo further treatment, while 30% of patients had chemotherapy alone and 30% had radiation alone. There was one patient (10%) who received both chemotherapy and radiation.

### 3.6. Recurrence-Free Survival

**Figure 2:**
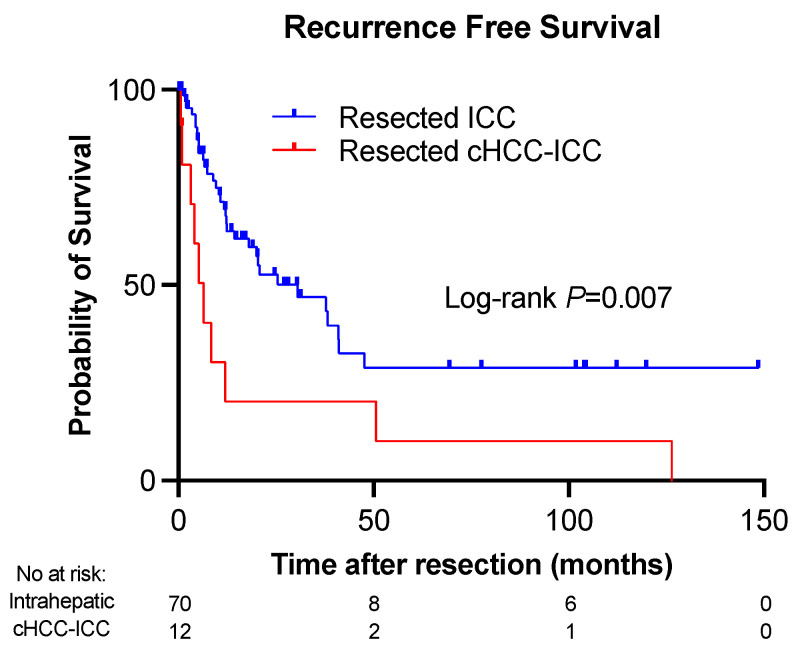
Worse recurrence-free survival among patients with cHCC-ICC. The number of subjects was 82. Intrahepatic in the figure = intrahepatic cholangiocarcinoma (ICC).

The recurrence-free survival (RFS) was significantly decreased among patients diagnosed with cHCC-ICC (log-rank *p* = 0.007), consistent with the previously stated higher rate of recurrence in this cohort; the RFS curves can be seen in Figure 2. Patients with ICC had a median RFS of 12.4 months (range 0.2–148.4 months), while patients with cHCC-ICC had a median RFS of 4.7 months (range 0.4–126.3). Although the cHCC-ICC group had a higher percentage of cirrhotic patients compared to the ICC group (33% vs. 4%), this did not seem to affect the finding that RFS was still significantly lower in non-cirrhotic patients (*p* = 0.008).

We then further stratified RFS by resection type. In patients with ICC, the median RFS in months was 19.8, 12.2, and 12.3 for extended, major, and minor resections, respectively (*p* = 0.763). In patients with cHCC-ICC, the median RFS was 3.7, 2, and 31.3 months for extended, major, and minor resections, respectively (*p* = 0.053).

### 3.7. Overall Survival and Mortality Rates

**Figure 3:**
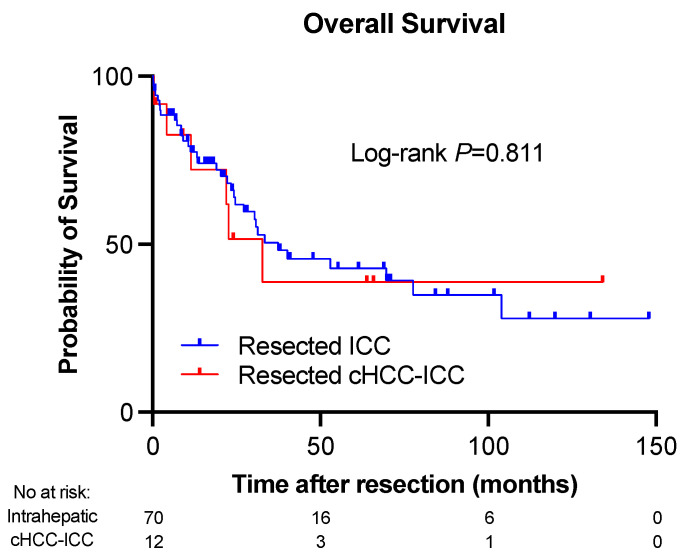
Similar overall survival between cHCC-ICC and ICC patients. The number of subjects was 82. Intrahepatic in the figure = intrahepatic cholangiocarcinoma (ICC).

Overall survival (OS) did not differ between resected ICC and cHCC-ICC as seen in Figure 3. OS of the entire group was 21.6 months (IQR 8.5–42.7 months). Patients diagnosed with ICC had a median OS of 21 months (range 0.2–147.8) while patients with cHCC-ICC had a median OS of 22.3 months (range 0.4–134). The respective 1-, 3-, and 5-year mortality rates for ICC patients were 23%, 51%, and 57%. For cHCC-ICC, the 1-, 3-, and 5-year mortality rates were 28%, 61%, and 61%, respectively.

As we had done for RFS, we stratified OS in the two separate groups by resection type. In the ICC group, the median OS was 22.8, 20.7, and 19 months for extended, major, and minor resections, respectively (*p* = 0.828). For cHCC-ICC, the median OS in months was 16.5, 7.8, and 49.3 for extended, major, and minor resections, respectively (*p* = 0.053).

### 3.8. Multivariate Analysis

The multivariable analysis of OS showed that patients with resected ICC had a 53% lower risk of death compared to those with resected cHCC-ICC; however, this difference was not statistically significant (HR 0.47 [0.15–1.47], *p* = 0.19). Conversely, the analysis of RFS demonstrated that patients with resected ICC had a significantly reduced risk of recurrence by 73% compared to their counterparts (HR 0.27 [0.10–0.73], *p* = 0.01). These results are shown in Table 4. 

## 4. Discussion

In this single-center retrospective study of 82 patients with either resected cHCC-ICC or ICC, we found significantly decreased RFS among patients with resected cHCC-ICC. This study contributes insight to the limited literature and studies currently published. Among the 82 resected patients, 70 patients were diagnosed with ICC, while 12 patients were diagnosed with cHCC-ICC. 

This study is among the first to report worse RFS in patients with resected cHCC-ICC. A Korean study evaluated the prognosis of cHCC-ICC in comparison to ICC in patients who underwent curative resection. For the cHCC-ICC group, the median time to recurrence (TTR) and OS were 5.4 and 18.0 months, respectively [29]. After adjusting for confounding factors, the cHCC-ICC group had a shorter TTR compared to the ICC group (RR, 2.00; *p* = 0.013). Similar to our study, the results of their study indicated that cHCC-ICC is associated with a significantly poorer prognosis than ICC after curative resection.

In a study by Penzkofer et al. they explored surgical outcomes in resected ICC and cHCC-ICC patients in Germany [25]. The study looked at a total of 202 ICC patients and 14 cHCC-ICC patients. Although the median RFS in patients with ICC appears to be shorter than in those with cHCC-ICC—7.3 months versus 16 months, respectively—the difference was not statistically significant (*p* = 0.479) [25]. In another Korean study, Lee et al. evaluated 79 resected ICC and 33 cHCC-ICC patients. In the study, unlike our results, they found worse clinical outcomes for patients with resected ICC compared to those with cHCC-ICC; the median disease-free survival after hepatic resection was 15.5 months for ICC patients versus 23.4 months for cHCC-ICC patients (*p* < 0.0001). In their study, portal vein invasion was found to be the only single significant predictor of a poor outcome after the hepatic resection of cHCC-ICC patients [30]. In our study, there was a trend toward a higher incidence of portal vein invasion in the cHCC-ICC group compared to the ICC group (42% vs. 16%, *p* = 0.051), which could partially explain the poorer clinical outcomes observed in the cHCC-ICC group.

Our study yielded results that differ from those reported in studies from Germany and Korea. This variation could potentially be partially due to geographic and racial differences, which may play roles in the disease’s manifestation and the resulting patient outcomes. The percentages of cirrhosis in the cHCC-ICC patients in our study and the studies by Penzkofer et al. and Lee et al. were 33%, 29%, and 48.5%, respectively. Additionally, our study had a significantly higher cHCC-ICC group with a history of hepatitis C, while the other studies primarily had patients with a history of Hepatitis B; this could be due to its higher prevalence in Asia, where those studies were conducted. One explanation for worse outcomes in cHCC-ICC in our cohort may be this patient group having a significantly higher rate of cirrhosis than the resected ICC group (4% vs. 33%, *p* = 0.008). Having different outcomes underscores the need for further research to elucidate the full spectrum of factors influencing these prognostic variations. However, given the rarity of cHCC-ICC, it is a limiting factor to conduct larger studies. 

In our cohort, patients with resected ICC had similar OS to that of resected cHCC-ICC. Leoni et al. recently published a comprehensive review of previous retrospective studies looking at OS for resected cHCC-ICC patients showing a wide range of OS from 16.5 to 52.5 months [24]. In the study by Penzkofer et. al., they found that there was also no significant OS difference between the cHCC-ICC and ICC groups. However, interestingly, it was found that the median OS was 17.6 months in the ICC group, while the median OS in the cHCC-ICC group was 26 months [25]. This contrasts with our study, where we found that the median OS was numerically higher in the cHCC-ICC group at 22.3 months compared to 21 months in the ICC group, which was not statistically significant. Several previous studies also found that there was no significant difference in OS between resected cHCC-ICC and ICC [29,30]. Similarly, a study by Yoon et al., which compared 53 cHCC-ICC patients and 149 ICC patients, did not find a significant difference in OS (8 vs. 6 months for cHCC-ICC and ICC, respectively) [31]. Contrary to those studies that found no difference in OS, a Surveillance, Epidemiology, and End Results (SEER)-based study by Yang et al. found that the OS for the cHCC-ICC group was significantly better than for the ICC group. One speculation that the study found to explain these findings is that the cHCC-ICC patients were diagnosed at an earlier stage [28]. The discrepancy in OS among these studies is likely explained by various factors, but one aspect is that many of the studies included patients who also underwent liver transplantation.

The utilization of tumor markers in cHCC-ICC has been debated, and several studies in the past have shown mixed conclusions. For example, one study suggested that the simultaneous elevation of both CA 19-9 and AFP may predict poorer outcomes [32]. Other studies have discussed the possible utility of tumor markers to aid in the differentiation of HCC and cHCC-ICC if both CA 19-9 and AFP are in concordance with imaging findings [5,33]. Although our study did not include HCC patients, we interestingly found significantly higher CA 19-9 values in the ICC group compared to the cHCC-ICC group, while AFP was significantly higher in the cHCC-ICC group. Given the rare occurrence of this disease, there are no well-defined guidelines on the management of these patients. Surgery continues to be the mainstay treatment in patients with resectable disease. However, in patients who are unresectable, the treatment options remain challenging and have poor outcomes. There have been several studies looking at HCC and ICC separately; however, there are limited data on therapeutic long-term outcomes in those with cHCC-ICC [24].

Recurrence rates remain a challenge in this patient population. In our study, we found a significantly higher rate of recurrence in patients diagnosed with cHCC-ICC (83% vs. 47%), which was also seen on multivariate analyses. However, there have been mixed findings in previous studies [34,35]. Similar to previous studies, intrahepatic recurrence was most frequently seen in both of our cohorts. In the resected ICC group, recurrence rates have been reported in the range of 61–73% in prior studies, which is slightly higher than our finding of 47% [36,37,38,39]. The slight difference in this could be accounted by the fact that some patients may have been lost to follow-up. For cHCC-ICC, previously reported rates of recurrence had a wider range, around 30–90% [24,29,40]. In our study, we found the rate of recurrence in our cHCC-ICC patients to be 83%. There can be a variety of factors to contribute to the increase rate of recurrence. One possible speculation is that, although not statistically significant, a higher percentage of patients in the resected ICC group had a major resection than the cHCC-ICC group. Additionally, however, a small number of the resected ICC patients, two, had a liver transplant. On further analysis, data on treatment after recurrence was not available in the majority of patients, but this could be an area of further exploration. Another potential explanation for higher rates of recurrence in the cHCC-ICC group could be a higher percentage of liver cirrhosis, which has been speculated in other studies as well [41].

cHCC-ICC exhibits unique biological features that may contribute to the poorer RFS observed in our study. This tumor type, which includes elements of both HCC and ICC, is characterized by significant cellular heterogeneity that may lead to a more aggressive disease course and resistance to conventional therapies. It was previously shown that cHCC-ICC genetics are distinct from those of ICC, which might be associated with increased tumor aggressiveness [42]. Our study does not include genomic information for the samples; therefore, it is unknown if this had any impact on the outcomes. Additionally, the tumor microenvironment in cHCC-ICC may further complicate recurrence outcomes. Furthermore, many cHCC-ICC patients have underlying liver cirrhosis, which was more common in our group compared to ICC. This may also provide a pro-tumorigenic environment that fosters recurrence. These mechanistic insights underscore the complex biology of cHCC-ICC and highlight the need for further research to improve outcomes in this challenging patient population.

Although our study contributes to the reporting of survival outcomes of the rare entity of cHCC-ICC, there are several limitations to our study. The main limitation is that, given the rare occurrence of cHCC-ICC patients, the small patient sample size can limit a generalization of the conclusions drawn from the study. Secondly, the possible inherent bias of a retrospective study could impact the conclusions drawn. Thirdly, selection bias associated with patients either referred or considered for surgical resection in either cohort can impact the inclusion of patients. Additionally, immunohistochemical (IHC) detection and pathology images were not readily available in our study. Lastly, our study did not include molecular profiling, nor was it large enough in the contemporary era to assess the impact of immunotherapy or other targeted therapies which now have a much more impactful benefit on survival outcomes for patients at the time of relapse. Despite these limitations, we believe that this study provides some groundwork to show that further studies need to be carried out in this patient population.

## 5. Conclusions

In summary, cHCC-ICC is a rare entity that needs to be further studied to improve the outcomes of our patients. Resection remains the only curative option for this patient population. Our study showed that RFS in the resected cHCC-ICC group was significantly lower than in the resected ICC group. This suggests that we need to perform more studies in this patient population, and perhaps a more aggressive initial approach, which may include systemic treatments such as chemoimmunotherapy to prevent recurrence, is warranted.

## Figures and Tables

**Table 1:** Resected Intrahepatic Cholangiocarcinoma vs. Combined Hepatocellular Carcinoma/Cholangiocarcinoma.

Characteristic	All patients (n = 82)	Resected Intrahepatic Cholangiocarcinoma (n = 70)	Resected cHCC-ICC (n = 12)	*p*-Value
Age, median (IQR), y	64 (56–69.5)	64 (56–72)	62 (54.25–66.75)	0.43
**Sex**				0.357
Male	43 (52%)	35 (50%)	8 (67%)	
Female	39 (48%)	35 (50%)	4 (33%)	
Hispanic or Latino	6 (7%)	5 (7%)	1 (8%)	0.999
**Race**				0.234
White	77 (94%)	66 (94%)	11 (92%)	
Black/African American	4 (5%)	4 (6%)	0	
Asian	1 (1%)	0	1 (8%)	
**History of prior cancer**				0.999
Yes	13 (16%)	11 (16%)	2 (17%)	
No	69 (84%)	59 (84%)	10 (83%)	
**Hepatitis B**	0	0	0	0.999
**Hepatitis C**	12 (15%)	7 (10%)	5 (42%)	**0.013**
**Cirrhosis**	7 (9%)	3 (4%)	4 (33%)	**0.008**
**ASA Classification**				0.061
ASA I	0	0	0	
ASA II	20 (24%)	20 (29%)	0	
ASA III	48 (59%)	38 (54%)	10 (83%)	
ASA IV	14 (17%)	12 (17%)	2 (17%)	
**Extended Resections**	8 (10%)	6 (9%)	2 (17%)	0.173
Right Trisectionectomy	4	4	0	
Left Trisectionectomy	3	1	2	
Mesohepatectomy	1	1	0	
**Major Resections**	58 (71%)	52 (74%)	6 (50%)	-
Right Hepatectomy	23	20	3	
Left Hepatectomy	20	17	3	
Trisegmentectomy	15	15	0	
**Minor Resections**	14 (17%)	10 (14%)	4 (33%)	-
Bisegmentectomy	7	7	0	
Monosegmentectomy	5	3	2	
Wedge Resections	2	0	2	
**Liver Transplant**	2 (2%)	2 (3%)	0	-
**CA 19-9 ˆ (U/mL)**	64.5 (16.75–278.5)	76 (20–423.6)	22 (5–64)	**0.02**
**Carcinoembryonic antigen (CEA) + (ng/mL)**	2.4 (1.5–3.7)	2.4 (1.5–3.75)	2.1 (1.2–3.91)	0.845
**Alpha Fetoprotein (AFP) ° (ng/mL)**	3.8 (2.2–6.45)	3.2 (1.85–5.4)	7.3 (4.23–19.75)	**0.0004**
**AST (U/L)**	62 (26.5–244.5)	46 (26–198) ‡	206 (83.25–566.8)	**0.012**
**ALT (U/L)**	57 (23.5–168)	48 (21.5–155.5) ‡	165.5 (68.5–403.8)	**0.013**
**Alkaline Phosphatase (U/L)**	87 (59.5–174.5)	104 (61.5–197.5) ‡	66 (55–86.75)	**0.03**
**Total Bilirubin (mg/dL)**	0.8 (0.5–1.8)	0.8 (0.45–2.15) ‡	1.05 (0.6–1.3)	0.785
**Neutrophil/Lymphocyte Ratio**	4.49 (2.51–10.25)	5.34 (2.82–11.46) ¥	2.99 (2.1–5.22)	**0.02**
**Vital Status**				0.999
Alive	42 (51%)	36 (51%)	6 (50%)	
Deceased	40 (49%)	34 (49%)	6 (50%)	
**Overall Survival (months)**	21.57 (8.52–42.67)	21.04 (8.52–42.67)	22.29 (5.42–56.05)	0.769

ˆ There are 13 intrahepatic cholangiocarcinoma patients and 3 cHCC-ICC patients missing CA 19-9 lab values. + There are 18 intrahepatic cholangiocarcinoma patients and 3 cHCC-ICC patients missing CEA lab values. ° There is 25 intrahepatic cholangiocarcinoma patients who are missing AFP lab values. ‡ There is one patient in the intrahepatic cholangiocarcinoma group without AST, ALT, alkaline phosphatase, or total bilirubin values. ¥ There were 6 patients without neutrophil and lymphocyte values available. -These boxes were left blank as the p-value in the Extended Resection Row is comparing extended resections with major, minor, and liver transplants. ASA: American Society of Anesthesiologists; CA 19-9: Carbohydrate antigen 19-9; AST: aspartate aminotransferase; ALT: alanine transaminase. Bolded *p*-Values in the last column indicate statistical significance.

**Table 2:** Histological Outcomes After Resection.

Characteristic	All Patients (n = 82)	Resected Intrahepatic Cholangiocarcinoma (n = 70)	Resected cHCC-ICC (n = 12) *	*p*-Value
**T Stage**				0.883
T1	20 (24%)	17 (24%)	3 (25%)	
T2	43 (52%)	36 (51%)	7 (58%)	
T3	12 (15%)	11 (16%)	1 (8%)	
T4	6 (7%)	6 (9%)	0	
**N Category**				0.999
N0	40 (49%)	34 (49%)	6 (50%)	
N1	10 (12%)	9 (13%)	1 (8%)	
Nx	31 (38%)	27 (39%)	4 (33%)	
**M Category**				0.999
M0	74 (90%)	63 (90%)	11 (92%)	
M1	8 (10%)	7 (10%)	1 (8%)	
**Tumor Size** (largest diameter in centimeters)	4.9 (3.08-8.78)	4.8 (3-8.4)	5.5 (3.4-9.7)	0.488
**Resection Status**				0.638
R0	70 (85%)	60 (86%)	10 (83%)	
R1	8 (10%)	6 (9%)	2 (17%)	
R2	4 (5%)	4 (6%)	0	
**Lymphovascular**				0.522
**Invasion**				
Yes	42 (51%)	35 (50%)	7 (58%)	
No	39 (48%)	35 (50%)	4 (33%)	
**Portal Vein Invasion**				**0.051**
Yes	16 (20%)	11 (16%)	5 (42%)	
No	66 (80%)	59 (84%)	7 (58%)	
**Perineural Invasion**				**0.007**
Yes	40 (49%)	39 (56%)	1 (8%)	
No	41 (50%)	31 (44%)	10 (83%)	

* There is one patient in the resected cHCC-ICC missing surgical histological data. Bolded *p*-Values in the last column indicate statistical significance.

**Table 3:** Location of Tumor Recurrence.

Characteristic	All Patients (n = 82)	Resected Intrahepatic Cholangiocarcinoma (n = 70)	Resected cHCC-ICC (n = 12)	*p*-Value
**Recurrence**	43 (52%)	33 (47%)	10 (83%)	**0.028**
**Recurrence Location**				0.999
Intrahepatic	27 (63%)	21 (64%)	6 (60%)	
Extrahepatic	9 (21%)	7 (21%)	2 (20%)	
Intrahepatic + Extrahepatic	7 (16%)	5 (15%)	2 (20%)	

Bolded *p*-Values in the last column indicate statistical significance.

**Table 4:** Cox Proportional Hazards Regression Multivariate Analysis of Survival.

Variable	Multivariate		
	HR	95% CI	*p*-Value
Overall Survival	0.47	0.15–1.47	0.19
Recurrence-Free Survival	0.27	0.10–0.73	**0.01**

Bolded *p*-Values in the last column indicate statistical significance.

## Data Availability

The data that support the findings of this study are available on request from the corresponding author.
